# Targeting the CSF1/CSF1R signaling pathway: an innovative strategy for ultrasound combined with macrophage exhaustion in pancreatic cancer therapy

**DOI:** 10.3389/fimmu.2024.1481247

**Published:** 2024-10-02

**Authors:** Qian Wang, Jianhong Wang, Ke Xu, Zhibin Luo

**Affiliations:** ^1^ Department of Ultrasound, Xichong People’s Hospital, Nanchong, China; ^2^ Department of Internal Medicine, Guang’an Vocational & Technical College, Guang’an, China; ^3^ Department of Oncology, Chongqing General Hospital, Chongqing University, Chongqing, China

**Keywords:** TAMs, immune exhaustion, TME, CSF1/CSF1R, ultrasound, pancreatic cancer

## Abstract

Pancreatic cancer (PC) is a highly aggressive and lethal malignancy characterized by a complex tumor microenvironment (TME) and immunosuppressive features that limit the efficacy of existing treatments. This paper reviews the potential of combining ultrasound with macrophage exhaustion in the treatment of pancreatic cancer. Macrophages, particularly tumor-associated macrophages (TAMs), are crucial in pancreatic cancer progression and immune escape. Prolonged exposure to the immunosuppressive TME leads to macrophage exhaustion, reducing their anti-tumor ability and instead promoting tumor growth. The CSF1/CSF1R signaling pathway is key in macrophage recruitment and functional regulation, making it an effective target for combating macrophage exhaustion. Ultrasound technology not only plays a significant role in diagnosis and staging but also enhances therapeutic efficacy by guiding radiofrequency ablation (RFA) and percutaneous alcohol injection (PEI) in combination with immunomodulators. Additionally, ultrasound imaging can monitor the number and functional status of TAMs in real-time, providing a basis for optimizing treatment strategies. Future studies should further investigate the combined use of ultrasound and immunomodulators to refine treatment regimens, address challenges such as individual variability and long-term effects, and offer new hope for pancreatic cancer patients.

## 1 Introduction

Pancreatic cancer is a highly aggressive and metastatic malignant tumor that is difficult to diagnose at an early stage and has an extremely poor prognosis (1–4). In recent years, the incidence and mortality of pancreatic cancer have continued to rise. Statistically, the 5-year survival rate for pancreatic cancer for the period 2013-2019 remains only 13%, one of the lowest among all cancers (4, 5). One of the main reasons for the poor prognosis is the lack of symptoms and inaccurate diagnosis in the early stages. About 90% of cases are diagnosed at an advanced stage, by which time more than 50% of patients have developed systemic metastases (5). The treatment of pancreatic cancer usually involves a multidisciplinary approach that includes chemotherapy, radiation, immunotherapy, and sometimes surgical interventions (3). However, the majority of pancreatic cancer patients who undergo radical resection and systemic chemotherapy still experience recurrence with local or systemic metastases (6). Commonly used chemotherapeutic agents such as gemcitabine and FOLFIRINOX have limited their application due to rapid chemoresistance (7–9). On the other hand, although emerging immunotherapies have demonstrated significant efficacy in a variety of cancers, their efficacy in pancreatic cancer and other quiescent tumors such as breast cancer remains limited (10, 11). Therefore, there is an urgent need to discover new therapies for pancreatic cancer patients, especially combination therapy strategies.

The TME of pancreatic cancer is complex and uniquely characterized, contributing to tumor immune escape and therapeutic failure (1). Among its components, TAMs play a crucial role in determining tumor growth, and therapeutic resistance (12). Prolonged exposure to cancer antigens and inhibitory TME significantly impairs macrophage efficacy, leading to their multifaceted functional exhaustion, including the upregulation of immune checkpoint ligands (13). In particular, the CSF1/CSF1R signaling pathway, which modulates TAM functions and behaviors, is vital in regulating the TME. Therefore, targeting the CSF1/CSF1R signaling pathway may represent a promising anti-tumor strategy in the future.

Ultrasound technology also has significant applications in the diagnosis and treatment of pancreatic cancer. Ultrasound-guided fine-needle aspiration (FNA) and coarse-needle biopsy (CNB) can accurately obtain macrophage samples from the TME, providing essential data for studying the role of TAMs in pancreatic cancer (14–16). Additionally, interventional techniques such as ultrasound-guided radiofrequency ablation (RFA) and percutaneous alcohol injection (PEI) can be combined with immunomodulators for precise delivery of targeted drugs, improving treatment efficacy (17, 18). Ultrasound can also monitor changes in the number and functional status of TAMs in real time during the treatment process, optimizing the treatment strategy (19, 20). Therefore, combining ultrasound with macrophage exhaustion and incorporating the unique properties of the TME may bring new breakthroughs and hope for pancreatic cancer patients.

## 2 Macrophage exhaustion

### 2.1 Classification and function of TAMs in TME

**Figure 1:**
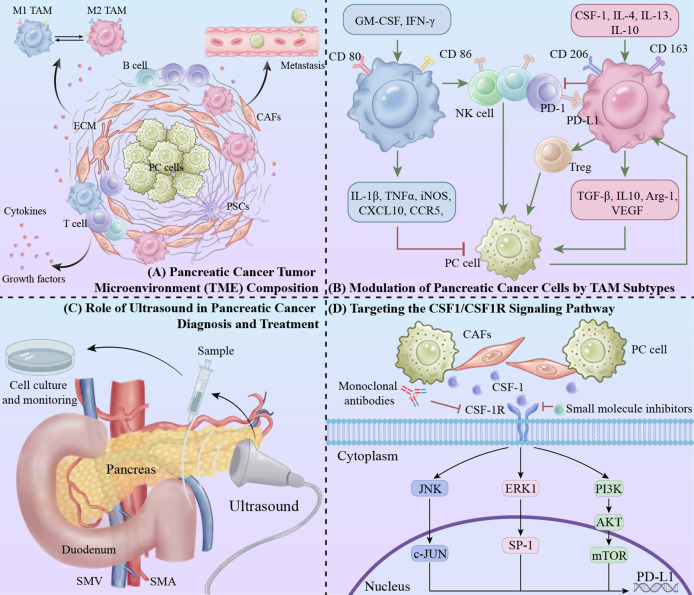
Integrative approaches in pancreatic cancer: targeting tumor microenvironment and macrophage modulation via ultrasound and CSF1/CSF1R pathway inhibition. **(A)** Pancreatic cancer TME composition. **(B)** Modulation of pancreatic cancer cells by TAM subtypes: The red “T” symbol indicates inhibitory effects, while the green arrow represents promotive actions within the signaling pathways. **(C)** Role of ultrasound in pancreatic cancer diagnosis and treatment. **(D)** Targeting the CSF1/CSF1R signaling pathway.

Pancreatic cancer has a highly reactive TME enriched with immunosuppressive and inflammatory cells capable of specifically silencing anti-tumor immune responses, allowing tumor cells to evade the effects of traditional monotherapies such as chemotherapy, immunotherapy, etc (1, 21). In addition, the TME of pancreatic cancer specifically contains pancreatic stellate cells (PSCs), cancer-associated fibroblasts (CAFs) as well as a variety of non-cellular components such as extracellular matrix (ECM), cytokines, and growth factors (1, 22). Together, these components contribute to an immunosuppressive TME, which ultimately leads to metastasis of tumor cells (**Figure 1A**).

Macrophages, as an important component of the innate and adaptive immune system, are widely studied immune cells in TME. The majority of macrophages originate from circulating monocytes. Based on their morphology, phenotype, and function, TAMs can be divided into 2 main classes: “anti-tumor” classically activated M1 TAMs and “pro-tumor” selectively activated M2 TAMs (**Figure 1B**) (12, 13). In the typical classification, pro-inflammatory macrophages acquire the M1 phenotype through activation of granulocyte-macrophage colony-stimulating factor (GM-CSF), lipopolysaccharide, and interferon gamma (IFN-γ), and anti-inflammatory macrophages acquire the M2 phenotype through induction of Colony-stimulating factor 1 (CSF-1), interleukin 4 (IL-4), IL-13, and IL-10 (23, 24). In the M2 TAM, there are characteristic biomarkers, including CDC, CDSF, and IL-10, that are associated with the M2 phenotype. characteristic biomarkers, including CD206 and CD163, among others. Biomarkers of M1 TAM polarization include CD80 and CD86. These molecules form the basis for the identification of TAMs (25).

### 2.2 Impact of macrophage exhaustion on pancreatic cancer development

Immune cell depletion is a dysfunctional state characterized by the fact that immune cells that play a key role in the antitumor immune response, such as T cells, natural killer (NK) cells, B cells, and macrophages together, exhibit reduced effector function. This depletion is not only a depletion of cell populations, but also manifests itself in altered expression of inhibitory receptors (e.g., PD-1, CTLA-4), metabolic dysregulation, and transcriptional changes leading to reduced cytokine production and proliferative capacity. TME plays a crucial role in this process. More research findings have been found for immune depletion targeting T cells, but it is still to be explored for the other immune cell depletion. Targeted macrophage exhaustion will perhaps be critical for the development of next-generation immunotherapies (26–28).

Prolonged exposure to immunosuppressive TME (characterized by low oxygen levels, high lactate levels, inflammation, and oxidative stress) significantly impairs the anti-inflammatory efficacy of macrophages, leading to macrophage exhaustion (29, 30). At this point, macrophages tend to adopt an M2 phenotype characterized by the secretion of immunosuppressive cytokines, such as IL-10, TGF-β, and other cytokines, in order to promote angiogenesis generation, lymphangiogenesis, immunosuppression, and tumor progression (13). macrophage exhaustion is also characterized by increased recruitment of M2 macrophages and upregulation of immune checkpoint ligands (e.g., PD-1L). Together, these changes promote immunosuppressive TME formation in pancreatic cancer patients, further leading to a vicious cycle.

In addition, data suggest that TAMs in immunosuppressive TME can also lead to depletion of other immune cells, particularly NK cells and T lymphocytes. TAMs can not only promote T cell depletion by overexpression of PD-1L, but also block cytotoxicity by expression of TAM-associated molecules, such as collagen structural macrophage receptor (MARCO) and CD163 T cell and natural killer cell activation (31). Deficiency of MARCO was found to significantly inhibit tumor progression and metastasis in a mouse model of pancreatic cancer, and correlation of clinical data showed a strong trend toward poorer survival in patients with high CD163 and MARCO macrophage infiltration (32). Notably, targeting these molecules with antibodies or knocking down these molecules may repolarize the TAM, thereby restoring the ability of T cells and anti-tumor capacity of natural killer cells, as well as down-regulate the activity of regulatory T cells (Treg) (33, 34). Considering that TAM induces immunosuppressive TME, reprogramming TAM to modulate the anti-tumor immune response has been suggested as a novel therapeutic approach for pancreatic cancer treatment.

## 3 CSF1/CSF1R signaling pathway

CSF-1, derived from fibroblasts, tumor cells, etc., is produced in membrane-bound form, secreted glycoproteins and proteoglycans. Currently, CSF-1R is considered to be the sole receptor for CSF-1. These cells regulate macrophage growth, differentiation and function by secreting CSF1. Colony-stimulating factor receptor (CSF1R), a type I single-transmembrane protein, is ubiquitously expressed in myeloid cells such as monocytes, macrophages, neuroglia, and osteoblasts. CSF1R induces receptor homodimerization by binding to either CSF-1 or IL-34, followed by activation of receptor signaling and activation of extracellular pro-cell-survival kinase cascades, including PI3K, ERK1/2, and JNK (35–38).

### 3.1 Role of CSF1/CSF1R in TME

The CSF1/CSF1R-mediated signaling pathway is critical for the differentiation and recruitment of the mononuclear phagocyte system, particularly macrophages. In TME, activation of the CSF1/CSF1R signaling pathway plays a critical role by promoting the transformation of macrophages to an immunosuppressive phenotype (i.e., M2-type macrophages) (39). These M2-type macrophages inhibit anti-tumor immune responses and promote tumor growth and metastasis by secreting a variety of immunosuppressive factors (e.g., IL-10, TGF-β) and factors that promote tumor angiogenesis (e.g., VEGF). In addition, the CSF1/CSF1R signaling pathway maintains the immunosuppressive state of the TME by regulating the recruitment and survival of TAMs (35).

CAFs are present in tumors at all stages, are heterogeneous, and their primary function is to synthesize, deposit, and remodel the ECM. However, CAFs also secrete cytokines, chemokines, growth factors, and angiogenic factors. In the TME, CAFs and TAMs can interact via the CSF1-CSF1R axis (40). For example, CSF1 expression was positively correlated with the abundance of CSF1R+ CD163+ macrophages in skin cancer patients, which is consistent with a role for CSF1 in mediating macrophage survival (41). In addition to fibroblasts, tumor cells can also secrete CSF1, suggesting that it may play a pro-tumorigenic role. Consistent with this, in metastatic PDAC, tumor cell-derived CSF1 induces macrophages to produce granulin, a secreted glycoprotein that promotes fibroblast activation and stimulates tumor growth (42).

Since the presence of CSF1R+ macrophages within tumors correlates with poor survival in various tumor types, targeting CSF1/CSF1R signaling pathway transduction that promotes tumor growth is an attractive strategy to eliminate TAMs, reduce M2 macrophage recruitment, or repolarize them (43).

### 3.2 Relationship between CSF1/CSF1R and macrophage exhaustion

The CSF1/CSF1R signaling pathway plays an important role in macrophage exhaustion, and its activation induces macrophage exhaustion, causing them to lose their anti-tumor function and instead support tumor growth and immune escape (44). Studies have shown that macrophage polarization in the TME is highly dependent on the presence of cytokines originating from the tumor cells, from other stromal cells (e.g., immune cells or fibroblasts), and from the local cytokine environment of the macrophages themselves. M2 TAM is the result of the persistence of growth factors such as CSF1 and cytokines such as IL-4 and IL-10 (45–47). M2 TAM, in addition to its direct tumor growth-promoting ability, suppresses immune effector cell function, thereby contributing to the elimination of tumor cells. This silencing of immune effector cells is achieved through the production of cytokines and enzymes that inhibit effector cells either directly or indirectly through other immune cell types such as intratumoral dendritic cells (DCs), Treg cells, and type 2 helper T cells (48–50).

In addition, over-activation of the CSF1/CSF1R signaling pathway not only leads to increased expression of macrophage surface inhibitory receptors, which further inhibits the function of immune cells, such as macrophages and T-cells, but also induces metabolic changes in macrophages, such as a decrease in oxidative phosphorylation and an increase in glycolysis, which can lead to their functional depletion (51–53).

## 4 Combination therapeutic strategies targeting macrophage exhaustion

### 4.1 Therapies targeting macrophage exhaustion

Therapeutic strategies targeting macrophage exhaustion focus on improving the suppressive TME and enhancing anti-tumor immune responses by inhibiting macrophage recruitment, reprogramming TAMs, and directly depleting TAMs. These strategies have shown promising potential in preclinical studies and are expected to play an important role in future cancer therapy.

The CSF1/CSF1R signaling pathway plays a key role in the recruitment and maintenance of TAMs. By inhibiting this signaling pathway, the recruitment of macrophages to the TME can be effectively reduced, decreasing their number and immunosuppressive function. CSF1R inhibitors such as Pexidartinib (PLX3397) have shown promising anti-tumor effects in preclinical and clinical studies (54).

TAMs in the TME usually behave as M2-type macrophages, which have immunosuppressive and tumor growth-promoting functions. Reprogramming TAMs to reverse-polarize them to M1-type macrophages can enhance their anti-tumor functions. Methods include the use of TLR agonists, IFN-γ and CSF1R inhibitors, etc. (55–59), which promote the production of pro-inflammatory cytokines and anti-tumor factors by altering the polarization state of macrophages to enhance the overall anti-tumor immune response.

Depletion of TAMs is another effective therapeutic strategy that can significantly improve the anti-tumor immune environment by directly targeting and removing these immunosuppressive cells. Targeted drugs commonly used today, such as anti-CSF1R antibodies, can induce apoptosis or functional inhibition of TAMs by blocking the CSF1/CSF1R signaling pathway (60). Cytotoxic drugs, such as Clodronate Liposomes, also deplete TAMs by inducing apoptosis (61).

### 4.2 Interventions targeting the CSF1/CSF1R signaling pathway

Interventions targeting the CSF1/CSF1R signaling pathway reduce the number and function of macrophages, improve the immunosuppressive state in the TME, and reverse immune depletion through multiple mechanisms (**Table 1**) (**Figure 1C**). In combination with other immunotherapies, it is expected to further improve the therapeutic efficacy and provide a new treatment strategy for pancreatic cancer patients (43, 62, 63).

**Table 1:** CSF1/CSF1R inhibitors as monotherapy in current clinical development.

Class	Compound	Action mechanism	Clinical stage	indication	Reference
Small molecule inhibitors	Pexidartinib(PLX3397)	By blocking the binding of CSF1 and CSF1R, the activation of downstream signaling pathway is inhibited	Phase III	Tenosynovial giant cell tumour (TGCT), various malignancies	(54, 64, 65)
	Sotuletinib(BLZ945)	Competes with the ATP-binding site of CSF1R, inhibiting its kinase activity and downstream signaling	Phase I/II	Advanced Solid Tumors	(66, 67)
	ARRY-382	The activity of CSF1R kinase was blocked and downstream signaling was inhibited	Phase I/II	Chronic lymphocytic leukemia (CLL), advanced or metastatic cancer	(68)
	Edicotinib(JNJ-40346527)	Blocking CSF1R kinase activity reduces macrophage survival and recruitment	Phase II	Alzheimer's disease, rheumatoid arthritis and HL (Hodgkin lymphoma)	(69, 70)
	Vimseltinib (DCC-3014)	The activity of CSF1R kinase was blocked and downstream signaling was inhibited	Phase III	TGCT	(71, 72)
	Sulfatinib (Surufatinib, HMPL-012)	Inhibition of tumor cell growth and angiogenesis through multi-target mechanisms	Phase III/Phase II	NET, thyroid cancer, biliary tract carcinoma and soft tissue sarcoma, SCLC, etc	(73–75)
Monoclonal antibodies	Emactuzumab	Blocking CSF1/CSF1R signaling, inducing receptor internalization and degradation, and killing macrophages through ADCC action	Phase I	Advanced/metastatic solid tumors, such as pancreatic cancer, or non-small cell lung cancer (NSCLC)	(76, 77)
	AMG 820	CSF1/CSF1R signal transduction is blocked to reduce the number of TAMs	Phase II	Advanced solid tumors	(78)
	Cabiralizumab (FPA008)	CSF1/CSF1R signaling was blocked, and macrophage recruitment and survival were inhibited	Phase II	RA (Rheumatoid arthritis), LC	(79)
	LY3022855	The activity of CSF1R kinase was blocked and downstream signaling was inhibited	Phase I	Advanced refractory breast or prostate cancer	(80)
	Axatilimab (SNDX-6352)	Blocking CSF1/CSF1R signaling can affect the migration, proliferation, differentiation and survival of monocytes and macrophages	Phase I	Chronic graft-versus-host disease (cGVHD) and tumor diseases	(81)

CSF1R inhibitors inhibit the activation of downstream signaling pathways by blocking the binding of CSF1 to CSF1R, thereby reducing macrophage survival, proliferation and recruitment to the TME. Currently, several CSF1R inhibitors have shown promising anti-tumor effects in clinical trials. For example, Pexidartinib (PLX3397) significantly reduced the number of TAMs and enhanced T-cell-mediated anti-tumor immune responses in multiple tumor models (82).

Monoclonal antibody targeting CSF1R is also an effective strategy.CSF1R antibodies kill CSF1R-expressing macrophages by blocking CSF1/CSF1R signaling, inducing receptor internalization and degradation, and by antibody-dependent cell-mediated cellular cytotoxicity (ADCC) effects. For example, Emactuzumab, a monoclonal antibody targeting the CSF1R, has demonstrated potential therapeutic efficacy in a variety of solid tumors (76, 77, 83). Bispecific antibodies are an emerging strategy for targeting both CSF1Rs and other tumor-associated antigens to enhance anti-tumor effects. Several studies are developing bispecific antibodies targeting CSF1R and PD-L1 to enhance therapeutic efficacy by simultaneously inhibiting immunosuppressive signaling and activating immune effects.

Small molecule inhibitors are also effective tools for targeting the CSF1R signaling pathway. These inhibitors block downstream signaling by competing with the ATP-binding site of CSF1R and inhibiting its kinase activity. For example, Sotuletinib(BLZ945), a potent small molecule inhibitor of CSF1R, has shown the ability to enhance immune responses in a variety of cancer models.

Combining inhibitors of the CSF1/CSF1R signaling pathway with other immunotherapies may further enhance therapeutic effects (84, 85). For example, in patients with advanced pancreatic cancer, CSF1R inhibitors in combination with PD-1/PD-L1 inhibitors may simultaneously deregulate immune checkpoint inhibition and reduce the number of immunosuppressive macrophages, thereby enhancing the anti-tumor response of T cells (86). In addition, CSF1R inhibitors may also be able to be used in combination with chemotherapy, radiotherapy, or other targeted therapies to enhance the tumor cell killing effect.

### 4.3 Combined ultrasound and macrophage exhaustion in an integrated treatment program

At the diagnostic and staging level, FNA and CNB are precise methods used for the diagnosis and staging of pancreatic cancer. These techniques accurately localize the tumor and the associated microenvironment through real-time imaging, obtaining cell and tissue samples from the tumor and its surrounding tissues (15, 16). These samples can be further used to analyze the status of macrophages in the TME, to understand their depletion and immunosuppressive properties, and to provide a basis for subsequent treatment.

On the other hand, at the therapeutic level targeting the TME, RFA and PEI performed under ultrasound guidance can effectively ablate tumor tissues while reducing immunosuppressive macrophages in the TME (17, 18). RFA ablates the tumor cells by thermal effects, while PEI by injecting ethanol causes cell dehydration and necrosis. Used in combination with immunomodulators, such as CSF1R inhibitors and anti-PD-1 antibodies, the macrophage exhaustion state can be further suppressed and the anti-tumor immune response enhanced.

In addition, ultrasonography can dynamically assess tumor blood flow and tissue properties, indirectly reflecting macrophage activity (19). Specifically, ultrasonography combined with nanotechnology for real-time *in situ* imaging of macrophages can directly and dynamically monitor changes in the number of TAMs in the TME (20). Through ultrasound monitoring, treatment plans can be promptly adjusted to effectively alleviate macrophage exhaustion, thereby enhancing overall treatment efficacy (**Figure 1D**).

## 5 Discussion

Although the therapeutic strategy of ultrasound combined with macrophage exhaustion has demonstrated significant potential in preclinical and clinical studies, some limitations remain, such as individual variability of treatment and unclear long-term effects on the TME in pancreatic cancer (87–89). Exploring different immunomodulator combinations with ultrasound is crucial to overcome patient variability. Optimizing dosage, managing side effects, and creating standardized protocols are essential for long-term treatment success (21).

Applying ultrasound combined with macrophage exhaustion in clinical settings presents challenges, such as determining optimal dosage, managing side effects, and establishing standardized protocols. Overcoming these challenges is crucial for clinical translation and improving treatment efficacy for pancreatic cancer patients. Evidence from other clinical applications, like ultrasound-guided hormone injections, shows enhanced treatment outcomes and supports the potential effectiveness of combining ultrasound with immunomodulatory therapies.

Macrophage exhaustion plays a critical role in tumor progression and immune suppression. Targeting and reversing macrophage exhaustion with ultrasound therapy can improve the TME and enhance anti-tumor immune responses (90). Future research should focus on increasing the specificity and efficacy of this strategy to address the complexity of pancreatic cancer. Overall, optimizing these protocols will improve patient outcomes and survival rates (91–93).
